# The structure of sperm Izumo1 reveals unexpected similarities with *Plasmodium* invasion proteins

**DOI:** 10.1016/j.cub.2016.06.028

**Published:** 2016-07-25

**Authors:** Kaoru Nishimura, Ling Han, Enrica Bianchi, Gavin J. Wright, Daniele de Sanctis, Luca Jovine

**Affiliations:** 1 Department of Biosciences and Nutrition & Center for Innovative Medicine, Karolinska Institutet, Huddinge, SE-141 83, Sweden; 2 Cell Surface Signalling Laboratory, Wellcome Trust Sanger Institute, Hinxton, Cambridge, CB10 1SA, UK; 3 ESRF - The European Synchrotron, Grenoble 38000, France

## Abstract

Fertilization, the culminating event in sexual reproduction, occurs when haploid sperm and egg recognize each other and fuse to form a diploid zygote. In mammals this process critically depends on the interaction between Izumo1, a protein exposed on the equatorial segment of acrosome-reacted sperm, and the egg plasma-membrane-anchored receptor Juno 1, 2. The molecular mechanism triggering gamete fusion is unresolved because both Izumo1 and Juno lack sequence similarity to known membrane fusogens. Here we report the crystal structure of Izumo1, which reveals a membrane distal region composed of a four-helix bundle connected to a carboxy-terminal immunoglobulin (Ig)-like domain through a β-hairpin stabilized by disulfide bonds. Remarkably, different regions of Izumo1 display significant structural similarities to two proteins expressed by the invasive sporozoite stage of *Plasmodium* parasites: SPECT1, which is essential for host cell traversal and hepatocyte invasion [3]; and TRAP, which is necessary for gliding motility and invasion [4]. These observations suggest a link between the molecular mechanisms underlying host cell invasion by the malaria parasite and gamete membrane fusion at fertilization.

## Teaser

Interaction between sperm Izumo1 and egg Juno is essential for triggering the fusion of mammalian gametes, but the sequences of these proteins do not resemble known membrane fusogens. Here, Nishimura *et al.* report the crystal structure of mouse Izumo1 and show that this shares similarities with *Plasmodium* proteins involved in host cell invasion.

## Main Text

**Figure 1:**
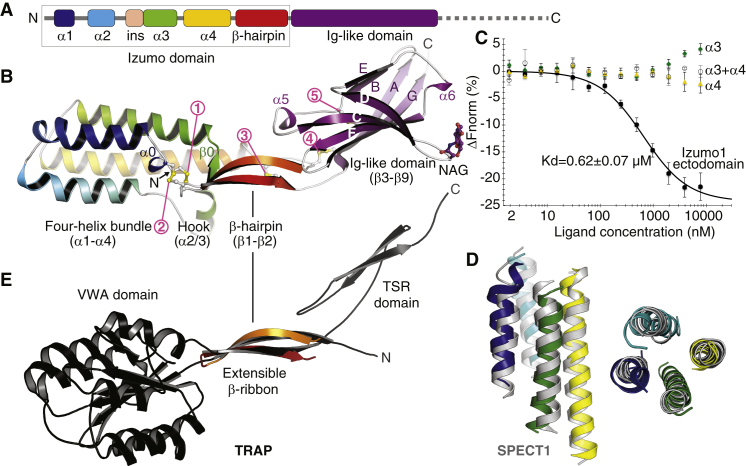
Experimental results and structural comparisons with *Plasmodium* invasion proteins. (A) Domain architecture of the extracellular region of Izumo1. The Izumo domain, which consists of a four-helix bundle (α1–α4), an insertion (ins: α2/3 hook and β0) and a β-hairpin (β1–β2) — see panel (B) — is boxed. A protease-sensitive carboxy-terminal region that was included in the expression construct used for this study, but is not defined in the electron density map, is indicated by a dashed line. (B) Crystal structure of mouse Izumo1 (amino acids C22–K256), shown in cartoon representation with different regions of the molecule colored as in (A). Amino/carboxyl termini and secondary structure elements are marked. Cysteine residues and the N-acetylglucosamine (NAG) residue attached to N204 are represented in ball-and-stick notation, with circled pink numbers indicating the five disulfide bonds (see also Figure S2A). (C) Microscale thermophoresis experiments show that Juno directly binds to the Izumo1 ectodomain, but does not interact with peptides corresponding to Izumo1 helices α3 and α4 (or an equimolar mixture thereof), which span a region reported to be sufficient for binding to the egg membrane [6]. Vertical error bars represent s.d. of the mean (n ≥ 3). (D) Structural alignment of the four-helix bundles of Izumo1 and *Plasmodium* SPECT1 (PDB ID 4U5A[8]), in side (left) and top (right) views. Izumo1 helices are colored as in (A,B), SPECT1 is grey. By optimizing the match found by Dali, 84 residues can be superimposed with a RMSD of 3.0 Å. (E) Superposition of the β-hairpin of Izumo1 (red) and the extensible β-ribbon of *Plasmodium* protein TRAP (black; PDB ID 4HQO[4]). Both elements separate amino-terminal α-helical domains from carboxy-terminal β-rich regions in the respective proteins.

Juno and Izumo are structurally unrelated: Juno adopts a modified folate receptor family fold [5], whereas Izumo1 is a type I transmembrane protein consisting of an extracellular region of about 300 residues and a short cytoplasmic tail [2]. The biological activity of Izumo1 depends on the amino-terminal half of its ectodomain — a region named the ‘Izumo domain’ because it is shared with the paralogous sperm proteins Izumo2, 3 and 4 [6]; this region is followed by a glycosylated Ig-like domain [2] (Figure 1A).

To investigate how Izumo1 might trigger gamete fusion, we expressed the entire ectodomain in mammalian cells and determined a crystal structure to 2.5 Å resolution (Figure S1 in Supplemental Information and PDB ID 5B5K). The structure shows that the Izumo domain consists of a four-helix bundle with a flexible α-helical hook/β-sheet insertion, followed by a β-hairpin that connects the bundle to a seven-stranded Ig-like domain (Figure 1A,B). These features result in an elongated architecture stabilized by five intramolecular disulfide bonds that are conserved in the three Izumo paralogs, and are clearly discernible within the sequence of Spaca6, another sperm surface protein recently reported to be required for gamete fusion [7] (Figure S2A,B). This suggests that the similarity between Izumo1 and Spaca6 is not limited to the carboxy-terminal Ig-like domain, which can be superimposed onto the canonical V-set Ig-like domain of human CD2 (PDB ID 1HNF) — a molecule mediating the adhesion of lymphocytes to antigen-presenting cells — with a root mean square deviation (RMSD) of 2.1 Å over 86 residues.

The helical character of the carboxy-terminal half of the Izumo domain, which corresponds to helices α3 and α4 in our structure (Figure 1A,B), was shown to be important for the function of Izumo1 [6]. The structure readily explains why mutation of leucine residues to proline interferes with this biological activity by disrupting the fold of the four-helix bundle (Figure S2A,C). However, binding assays using peptides representing α3 and α4 suggest that this region of Izumo1 is either not involved in binding to Juno or not sufficient for this interaction (Figure 1C).

Strikingly, structural homology searches revealed a high confidence match between the four-helix bundle of Izumo1 and that of SPECT1, a secreted *Plasmodium* protein that is essential for host cell traversal by the infective sporozoite form of the parasite (Dali Z-score 6.9, RMSD 3.3 Å over 98 residues; Figure 1D). The nearly parallel/antiparallel arrangement of the SPECT1 helical bundle, which also contains a helical hook (albeit inserted at a different position than in Izumo1), has been suggested to adopt a metastable structure which favors the transition from a solvent-exposed to membrane-associated form [8]. In addition, the β-hairpin of Izumo1 is structurally similar to the extensible β-ribbon of TRAP, another *Plasmodium* protein that mediates sporozoite gliding and host cell invasion [4] (RMSD 1.4 Å over 19 residues; Figure 1E). The TRAP β-ribbon is thought to undergo conformational changes upon attachment to host cells and acts as an elbow-like flexible spacer between the amino-terminal α-helical von Willebrand factor A (VWA) domain of the protein (which interacts with the host cell) and its carboxy-terminal β-rich thrombospondin type I repeat (TSR) domain.

What might be the functional implications of the structural similarities to *Plasmodium* cell traversal and invasion proteins with Izumo1? Our findings suggest that the four-helix bundle of the Izumo domain and SPECT1 may orchestrate analogous fusion-related molecular recognition events at the sperm–egg and parasite–host cell interfaces. Izumo1 is not considered a fusogen per se because it cannot induce membrane fusion when heterologously expressed in commonly used cell lines [1]. The presence of solvent-exposed bulk aromatic residues in the Izumo1 four-helix bundle (Figure S2A; a feature also observed in SPECT1) and the report that some monoclonal antibodies that target the Izumo domain do not block binding of sperm to the egg but hinder gamete fusion [6] are consistent with the possibility that an additional, as yet unknown, component is required to form a functional fusogenic complex [9]. Whether SPECT1 is also part of a larger protein complex that could organize proteins that have membrane-disrupting functions, such as SPECT2, is currently unclear [10].

Both SPECT1 and TRAP are expressed by the sporozoite stage of *Plasmodium* parasites and are similarly sequestered within specialized intracellular secretory organelles called micronemes 3, 4. We do not yet know if these proteins, like Izumo1, have a specific binding partner that mediates cell-specific recognition events, or, as has been suggested for sp18 (a five-helical bundle sperm acrosomal protein from the mollusk abalone), are disrupting membrane structure by interacting directly with phospholipids. Nevertheless, it is intriguing that, similar to SPECT1 and TRAP, Izumo1 is sequestered in the acrosome and that its release is also highly regulated by localized secretion.

The structure of Izumo1 provides new mechanistic insights into a fascinating basic biological process and, also considering the protein’s recent implication in human immunoinfertility, presents new opportunities in the rational design of new fertility treatments and contraceptives.

## Supplemental Information

Document S1. Two Figures and Experimental Procedures
