# Supplementary material for: The structure of sperm Izumo1 reveals unexpected similarities with Plasmodium invasion proteins
Source: Curr Biol. 2016 Jul 25;26(14):R661–2. doi: 10.1016/j.cub.2016.06.028 (PMC4963209; doi:10.1016/j.cub.2016.06.028)
Supplement: Document S1. Two Figures and Experimental Procedures [file mmc1.pdf]

Supplemental Information

The structure of sperm Izumo1 reveals unexpected similarities with *Plasmodium* invasion proteins

Kaoru Nishimura, Ling Han, Enrica Bianchi, Gavin J. Wright, Daniele de Sanctis and Luca Jovine

A

**Data Collection**	
Wavelength (Å)	0.984
Resolution (Å)	42.3–2.5 (2.59–2.50)
Space group	*I* 4 2 2 (97)
Unit cell (Å, deg)	103.28, 103.28, 139.27, 90, 90, 90
Total reflections	168538 (13122)
Unique reflections	13389 (1304)
Multiplicity	12.6 (10.1)
Completeness (%)	100.0 (100.0)
Mean I/sigma(I)	12.1 (1.1)
Wilson B factor ($Å^2$)	59.20
R_{pim} (%)	5.7 (75.2)
CC(1/2)	1.00 (0.46)
CC*	1.00 (0.79)
**Refinement**	
Reflections	13383 (1302)
Free reflections	670 (65)
R_{work} (%)	25.73 (40.20)
R_{free} (%)	28.19 (44.22)
CC_{work}	0.92 (0.45)
CC_{free}	0.88 (0.34)
Number of non–H atoms	1960
Macromolecule	1917
Ligand	15
Water	28
Protein residues	235
RMS deviations	
Bond lengths (Å)	0.004
Bond angles (°)	0.82
Ramachandran plot	
Favored (%)	96.1
Allowed (%)	3.9
Outliers (%)	0
Rotamer outliers (%)	0
Clashscore	4.44 (99th percentile (N=271, 2.50 Å ± 0.25 Å)
MolProbity score	1.52 (99th percentile (N=6960, 2.50 Å ± 0.25 Å)
Average B factor ($Å^2$)	59.93
Macromolecule	59.58
Ligand	61.04
Water	83.33

B

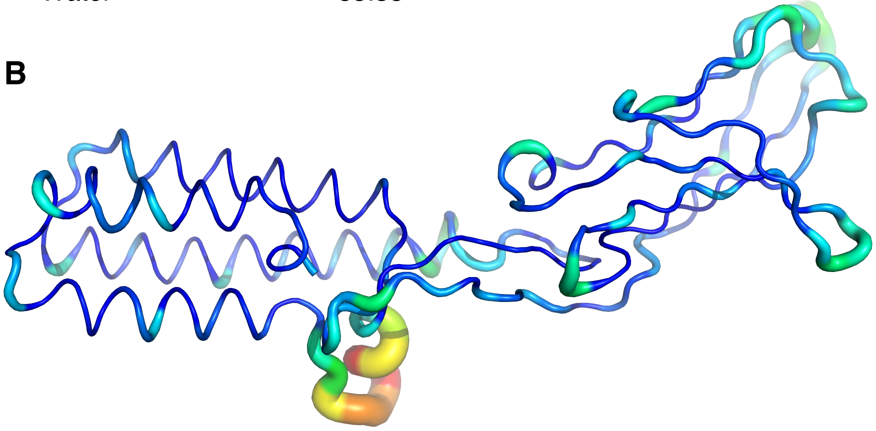

Figure S1. Structure determination of mouse Izumo1.

(A) X-ray data collection and refinement statistics. Values for the outermost shell are shown in parentheses. 100th percentile is the best among structures of comparable resolution, 0th percentile is the worst (for clashscore the comparative set of structures was selected in 2004, for MolProbity score in 2006). (B) Cα atom temperature factor distribution shown in B-factor "putty" representation, using a ribbon radius that increases from lowest (44.09 $Å^2$; dark blue) to highest (146.83 $Å^2$; red) B-factor. With the exception of the α-helical hook region (residues V66-G76), which has an average B-factor of 104 $Å^2$, the bulk of the Izumo1 structure (residues C22-G65, A77-K256) has relatively low and more uniform B-factors (average B-factor 56 $Å^2$). This suggests that, except for the flexible α-helical hook, highly intramolecularly disulfide-bonded Izumo1 is a rigid molecule.

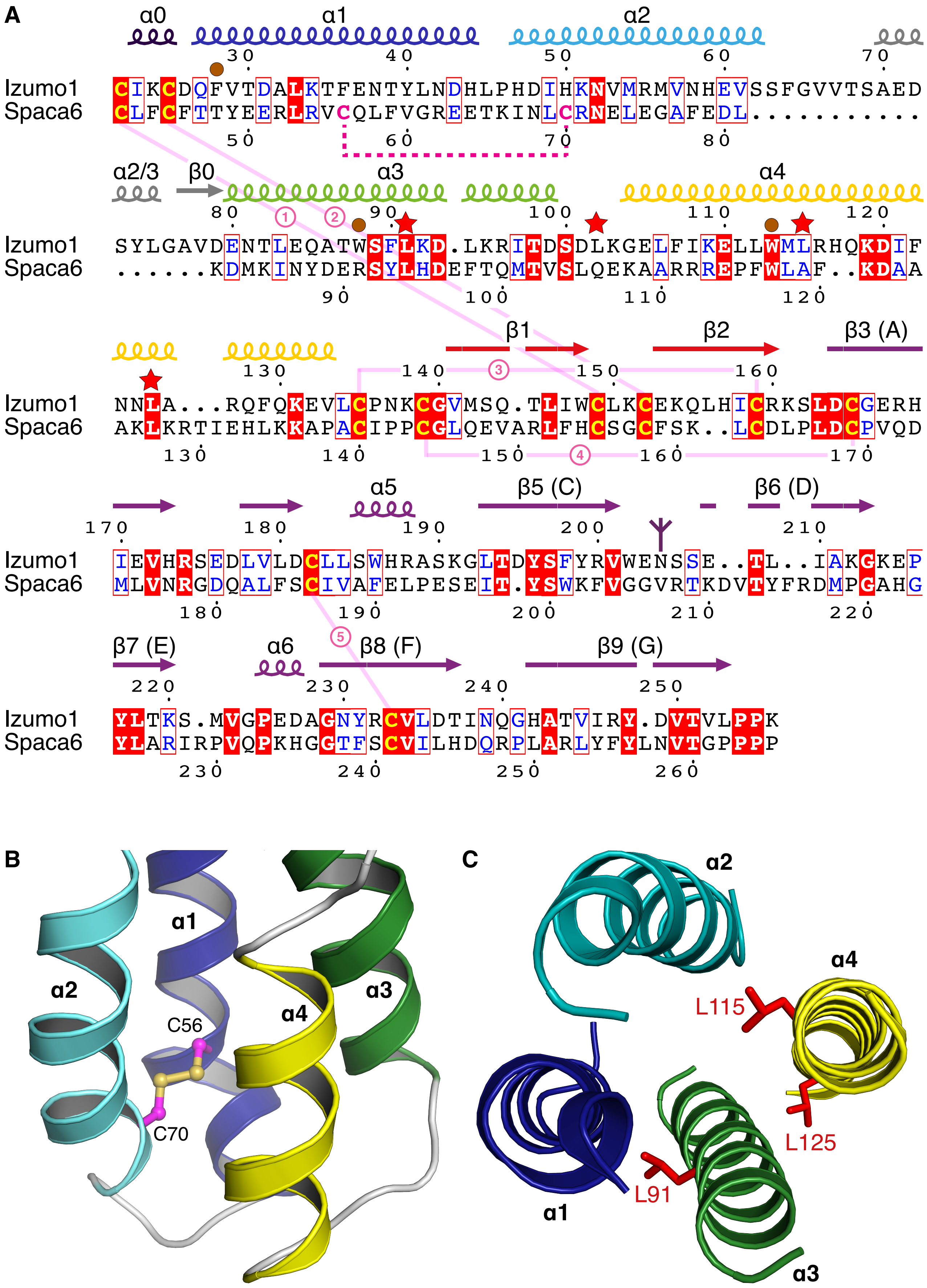

A
α0
α1
α2
Izumo1 CIKCDQFVTDALKTFENTYLNDHLPHDIHKNVMRMVNHEVSSFGVVTSAED
Spaca6 CLFCFTTYEERLRVCQLFVGREETKINLCRNELEGAFEDL...........
α2/3
β0
α3
α4
Izumo1 SYLGAVDENTLEQATWSFLKD.LKRITDSDLKGELFIKELLWMLRHQKDIF
Spaca6 ......KDMKINYDERSYLHDEFTQMTVSLQEKAARRREPFWLAF..KDAA
β1
β2
β3 (A)
Izumo1 NNLA...RQFQKEVLCPNKCGVMSQ.TLIWCLKCEKQLHICRKSLDCGERH
Spaca6 AKLKRTIEHLKKAPACIPPCGLQEVARLFHCSGCFSK..LCDLPLDCPVQD
α5
β5 (C)
β6 (D)
Izumo1 IEVHRSEDLVLDCLLSWHRASKGLTDYSFYRVWENSSE..TL..IAKGKEP
Spaca6 MLVNRGDQALFSCIVAFELPESEIT.YSWKFVGGVRTKDVTYFRDMPGAHG
β7 (E)
α6
β8 (F)
β9 (G)
Izumo1 YLTKS.MVGPEDAGNYRCVLDTINQGHATVIRY.DVTVLPPK
Spaca6 YLARIRPVQPKHGGTFSCVILHDQRPLARLYFYLNVTGPPPP
B
α2
α1
α4
α3
C56
C70
C
α2
α4
L115
L125
α1
L91
α3

Figure S2. The structural features of Izumo1 are conserved in sperm protein Spaca6. (A) Structure-based sequence alignment of mouse Izumo1 and Spaca6. identical amino acids are white in red boxes, except for cysteine residues that are highlighted in yellow; similar amino acids are blue in white boxes. Secondary structure elements of Izumo1, indicated above the sequences, are labelled and colored as in Figure 1A,B. Conserved disulfide bonds 1-5 are indicated by pink solid lines, and a sixth possible disulfide involving two additional cysteines of Spaca6 (C56 and C70, magenta) is indicated by a dashed magenta line (see also panel (B)). The single N-glycan of Izumo1 is represented by an inverted purple tripod. Izumo domain leucine residues whose combined mutation to proline impairs Izumo1 function ([S1]; panel (C)) are marked by red stars. Solvent-exposed aromatic residues in the four-helix bundle of Izumo1 are indicated by brown circles. (B) Homology modelling shows that the Izumo1 fold is compatible with an additional disulfide bond being formed between C56 and C70 of Spaca6. (C) The structure of Izumo1 clearly shows how the helical bundle fold would be disrupted upon introduction of multiple helix-breaking mutations affecting three leucine residues (red sticks), as well as L102 in the loop between α3 and α4 (not shown) [S1].

SUPPLEMENTAL EXPERIMENTAL PROCEDURES

Protein expression

A cDNA fragment encoding the mouse Izumo1 signal peptide and ectodomain (residues M1-P312) was subcloned into pHLsec3, a mammalian expression vector derived from pHLsec [S2], in frame with a 3' sequence for a LEH_6 affinity tag. The protein was expressed in glycosylation-defective human embryonic kidney 293S cells [S3, S4] (ATCC CRL-3022), deglycosylated with Endoglycosidase H and purified essentially as described [S5, S6].

Protein crystallization and X-ray diffraction data collection

Thin square plate crystals were obtained by sitting drop vapor diffusion at room temperature, using a mother liquor containing 20% (w/v) PEG 3350, 0.2 M ammonium formate pH 6.6. For data collection at 100 K, specimens were cryoprotected using mother liquor supplemented with 10% (v/v) PEG 200, mounted in MicroLoops (MiTeGen) and flash cooled in liquid nitrogen. Data was collected from a single 50 x 50 x 10 µm crystal at European Synchrotron Radiation Facility (ESRF) beamline ID29 [S7], using a PILATUS 6M-F detector (DECTRIS). Integration and scaling were performed with XDS [S8], according to described high resolution cutoff criteria [S9].

Structure determination and refinement

The structure was solved by molecular replacement with Phaser [S10], using the coordinates of human IZUMO1 (PDB ID 5JK9; kindly provided before publication by Prof. Toshiyuki Shimizu, University of Tokyo) as a search model. A single solution was obtained in space group *I*422, with one molecule per asymmetric unit (translation function Z-score: 11.5; log likelihood gain: 86). Correctness of the solution, which packed without any clash, was confirmed by MR-SAD (Molecular Replacement combined with Single-wavelength Anomalous Diffraction phasing) with a dataset collected at λ=1.35 Å from a crystal soaked for 46 h in 0.5 mM uranyl acetate (PHENIX AutoSol BAYES-CC: 53.8 +/- 19.8; FOM: 0.53; overall model-map correlation (101 residues): 0.497; R_{work}/R_{free}: 43%/50%) [S11]. Model building and refinement were performed with Coot [S12] and phenix.refine [S13], using feature-enhanced maps in addition to conventional σA-weighted maps [S14]. Protein geometry was validated with MolProbity [S15], carbohydrate structure validation was carried out using Privateer [S16]. Structure comparisons were performed using the Dali server [S17] and/or the sequence-independent structure-based dynamic programming alignment of PyMOL (Schrödinger); the latter was also used to optimize the superimposition with the Dali SPECT1 hit (Figure 1D). Structural figures were made with PyMOL; the alignment in Figure S2A was prepared using ESPript [S18]. Data collection and refinement statistics are summarized in Figure S1A. Structure factor intensities and atomic coordinates have been deposited in the Protein Data Bank with ID 5B5K.

Homology modeling

A homology model of mouse Spaca6 ectodomain residues C42-P265 [S19] was generated using MODELLER [S20] and energy minimized using YASARA Structure [S21].

Binding affinity determination by microscale thermophoresis

Microscale thermophoresis (MST) analysis [S22] was performed using a NanoTemper Monolith NT.115 instrument (NanoTemper Technologies GmbH). Mouse Juno was fluorescently labelled with a Blue-NHS labelling kit (NanoTemper Technologies GmbH), according to the manufacturer's instructions. The molar ratio of dye and protein was 4:1. Varying concentrations of mouse Izumo1 ectodomain (residues C22-P312, followed by a LEH_6 C-terminal affinity tag) or peptides corresponding to Izumo1 residues D79-K103 and/or G104-L134 (Thermo Fisher Scientific) were titrated against labelled mouse Juno (83 nM) in 20 mM Na-HEPES pH 7.8, 200 mM NaCl, 0.05% (v/v) Tween 20. Samples were loaded into Standard Treated Capillaries (NanoTemper Technologies GmbH) and MST measurements were performed using 20% MST laser power and 20% LED power. Laser-on and -off times were 30 seconds and 5 seconds, respectively. For each set of binding experiments, at least three independent MST measurements were carried out at 495 nm. Datasets were processed with the NanoTemper Analysis software, using the thermophoresis with T jump signal.

SUPPLEMENTAL REFERENCES

S1. Inoue N., Hamada D., Kamikubo H., Hirata K., Kataoka M., Yamamoto M., Ikawa M., Okabe M., and Hagihara Y. (2013). Molecular dissection of IZUMO1, a sperm protein essential for sperm-egg fusion. Development *140*, 3221-3229.

S2. Aricescu A.R., Lu W., and Jones E.Y. (2006). A time- and cost-efficient system for high-level protein production in mammalian cells. Acta Crystallogr. D Biol. Crystallogr. *62*, 1243-1250.

S3. Chang V.T., Crispin M., Aricescu A.R., Harvey D.J., Nettleship J.E., Fennelly J.A., Yu C., Boles K.S., Evans E.J., Stuart D.I. et al. (2007). Glycoprotein structural genomics: solving the glycosylation problem. Structure *15*, 267-273.

S4. Reeves P.J., Callewaert N., Contreras R., and Khorana H.G. (2002). Structure and function in rhodopsin: high-level expression of rhodopsin with restricted and homogeneous N-glycosylation by a tetracycline-inducible N-acetylglucosaminyltransferase I-negative HEK293S stable mammalian cell line. Proc. Natl. Acad. Sci. USA *99*, 13419-13424.

S5. Bokhove M., Sadat Al Hosseini H., Saito T., Dioguardi E., Gegenschatz-Schmid K., Nishimura K., Raj I., de Sanctis D., Han L., and Jovine L. (2016). Easy mammalian expression and crystallography of maltose-binding protein-fused human proteins. J. Struct. Biol. *194*, 1-7.

S6. Han L., Nishimura K., Sadat Al Hosseini H., Bianchi E., Wright G.J., and Jovine L. (2016). Divergent evolution of vitamin B_9 binding underlies Juno-mediated adhesion of mammalian gametes. Curr. Biol. *26*, R100-R101.

S7. de Sanctis D., Beteva A., Caserotto H., Dobias F., Gabadinho J., Giraud T., Gobbo A., Guijarro M., Lentini M., Lavault B. et al. (2012). ID29: a high-intensity highly automated ESRF beamline for macromolecular crystallography experiments exploiting anomalous scattering. J. Synchrotron Radiat. *19*, 455-461.

S8. Kabsch W. (2010). XDS. Acta Crystallogr. D Biol. Crystallogr. *66*, 125-132.

S9. Evans P.R., and Murshudov G.N. (2013). How good are my data and what is the resolution? Acta Crystallogr. D Biol. Crystallogr. *69*, 1204-1214.

S10. McCoy A.J., Grosse-Kunstleve R.W., Adams P.D., Winn M.D., Storoni L.C., and Read R.J. (2007). Phaser crystallographic software. J. Appl. Crystallogr. *40*, 658-674.

S11. Terwilliger T.C., Adams P.D., Read R.J., McCoy A.J., Moriarty N.W., Grosse-Kunstleve R.W., Afonine P.V., Zwart P.H., and Hung L.W. (2009). Decision-making in structure solution using Bayesian estimates of map quality: the PHENIX AutoSol wizard. Acta Crystallogr. D Biol. Crystallogr. *65*, 582-601.

S12. Emsley P., Lohkamp B., Scott W.G., and Cowtan C. (2010). Features and development of Coot. Acta Crystallogr. D Biol. Crystallogr. *66*, 486-501.

S13. Afonine P.V., Grosse-Kunstleve R.W., Echols N., Headd J.J., Moriarty N.W., Mustyakimov M., Terwilliger T.C., Urzhumtsev A., Zwart P.H., and Adams P.D. (2012). Towards automated crystallographic structure refinement with phenix.refine. Acta Crystallogr. D Biol. Crystallogr. *68*, 352-367.

S14. Afonine P.V., Moriarty N.W., Mustyakimov M., Sobolev O.V., Terwilliger T.C., Turk D., Urzhumtsev A., and Adams P.D. (2015). FEM: feature-enhanced map. Acta Crystallogr. D Biol. Crystallogr. *71*, 646-666.

S15. Chen V.B., Arendall W.B., Headd J.J., Keedy D.A., Immormino R.M., Kapral G.J., Murray L.W., Richardson J.S., and Richardson D.C. (2010). MolProbity: all-atom structure validation for macromolecular crystallography. Acta Crystallogr. D Biol. Crystallogr. *66*, 12-21.

S16. Agirre J., Iglesias-Fernandez J., Rovira C., Davies G.J., Wilson K.S., and Cowtan K.D. (2015). Privateer: software for the conformational validation of carbohydrate structures. Nat. Struct. Mol. Biol. *22*, 833-834.

S17. Holm L., and Rosenstrom P. (2010). Dali server: conservation mapping in 3D. Nucleic Acids Res. *38*, W545-9.

S18. Gouet P., Robert X., and Courcelle E. (2003). ESPript/ENDscript: Extracting and rendering sequence and 3D information from atomic structures of proteins. Nucleic Acids Res. *31*, 3320-3323.

S19. Lorenzetti D., Poirier C., Zhao M., Overbeek P.A., Harrison W., and Bishop C.E. (2014). A transgenic insertion on mouse chromosome 17 inactivates a novel immunoglobulin superfamily gene potentially involved in sperm-egg fusion. Mamm. Genome *25*, 141-148.

S20. Webb B., and Sali A. (2014). Comparative Protein Structure Modeling Using MODELLER. Curr. Protoc. Bioinformatics *47*, 5.6.1-32.

S21. Krieger E., Joo K., Lee J., Lee J., Raman S., Thompson J., Tyka M., Baker D., and Karplus K. (2009). Improving physical realism, stereochemistry, and side-chain accuracy in homology modeling: Four approaches that performed well in CASP8. Proteins *77 Suppl 9*, 114-122.

S22. Wienken C.J., Baaske P., Rothbauer U., Braun D., and Duhr S. (2010). Protein-binding assays in biological liquids using microscale thermophoresis. Nat. Commun. *1*, 100.

AUTHOR CONTRIBUTIONS

Conceptualization, K.N., G.J.W, L.J.; methodology, K.N., L.H., D.d.S., L.J.; validation, L.H.; formal analysis, K.N., L.H., D.d.S., L.J.; investigation, K.N., L.H., D.d.S., L.J.; data curation, K.N., L.J.; writing, K.N., G.J.W. and L.J., with comments from L.H, E.B. and D.d.S.; visualization, K.N., L.H., L.J.; supervision, G.J.W, L.J.; project administration, K.N., L.H., L.J.; funding acquisition, G.J.W, L.J.

NOTE ADDED IN PROOF

After acceptance of the present manuscript, two reports describing structural studies of human IZUMO1, JUNO and their complex were published (Aydin *et al.* (2016) Nature *534*, 562-565; Ohto *et al.* (2016) Nature *534*, 566-569).
